# Supplementary material for: Exome sequencing of familial high-grade serous ovarian carcinoma reveals heterogeneity for rare candidate susceptibility genes
Source: Nat Commun. 2020 Apr 2;11:1640. doi: 10.1038/s41467-020-15461-z (PMC7118163; doi:10.1038/s41467-020-15461-z)
Supplement: Supplementary file 1 — Supplementary Information [file 41467_2020_15461_MOESM1_ESM.pdf]

# Exome sequencing of familial high-grade serous ovarian carcinoma reveals heterogeneity of rare candidate susceptibility genes

## Supplementary Figure 1

*Subramanian et al.*

## Supplementary Figure 1

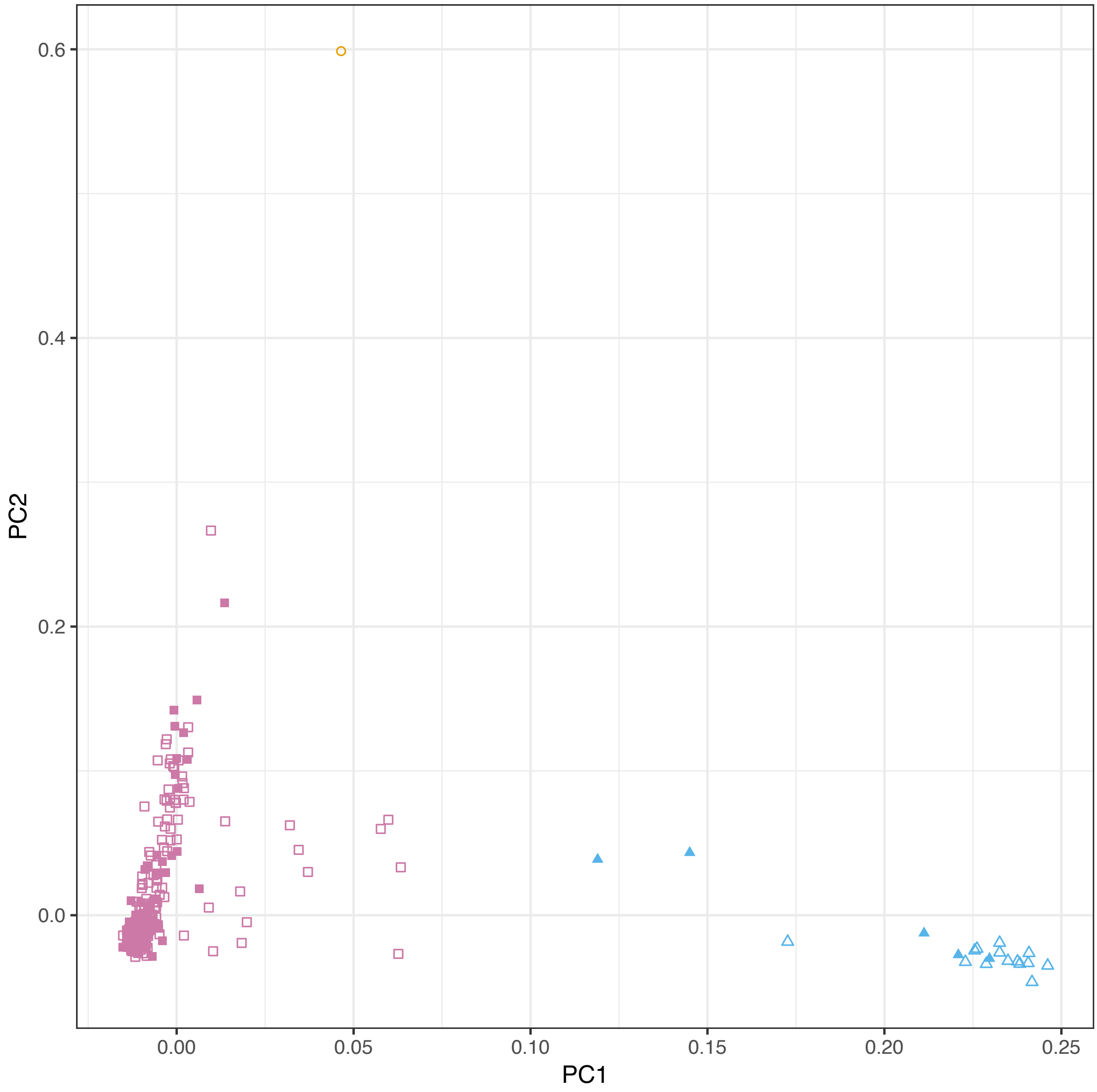


Dimensionality reduction of exomes (n = 516) using principal component analysis (PCA), with first (PC1) and second (PC2) principal component projections plotted on each axis. The pink squares, orange circles and blue triangles represent cases with estimated European, African and Asian ancestry, respectively. Filled and empty symbols represent samples with (n = 138) and without (n = 378) LoF variants from the candidate gene list (Table 4), respectively.
