# Supplementary material for: Exome sequencing of familial high-grade serous ovarian carcinoma reveals heterogeneity for rare candidate susceptibility genes
Source: Nat Commun. 2020 Apr 2;11:1640. doi: 10.1038/s41467-020-15461-z (PMC7118163; doi:10.1038/s41467-020-15461-z)
Supplement: Supplementary file 3 — Description of Additional Supplementary Files [file 41467_2020_15461_MOESM3_ESM.pdf]

Description of Additional Supplementary Files

File Name: Supplementary Data 1

Description: All genes (n = 4863) with 'HIGH' impact variants (n = 6674) in the case cohort (n= 510) that passed basic quality and frequency filters (Figure 1) and had no pathogenic variants in known ovarian carcinoma predisposition genes, including calculated Fisher's exact test results and risk ratios.

File Name: Supplementary Data 2

Description: Top-ranked 43 candidate genes with LoF variants in the case cohort that passed additional filters and manual curation (Figure 1), including annotated individual variants and anonymised case data.
