# Supplementary material for: Exome sequencing of familial high-grade serous ovarian carcinoma reveals heterogeneity for rare candidate susceptibility genes
Source: Nat Commun. 2020 Apr 2;11:1640. doi: 10.1038/s41467-020-15461-z (PMC7118163; doi:10.1038/s41467-020-15461-z)
Supplement: Supplementary file 6 — Reporting Summary. [file 41467_2020_15461_MOESM6_ESM.pdf]

nature research

Corresponding author(s): Deepak N. Subramanian

Last updated by author(s): Jan 30, 2020

Reporting Summary

Nature Research wishes to improve the reproducibility of the work that we publish. This form provides structure for consistency and transparency in reporting. For further information on Nature Research policies, see Authors & Referees and the Editorial Policy Checklist.

Statistics

For all statistical analyses, confirm that the following items are present in the figure legend, table legend, main text, or Methods section.

n/a	Confirmed	
☐	☒	The exact sample size (*n*) for each experimental group/condition, given as a discrete number and unit of measurement
☐	☒	A statement on whether measurements were taken from distinct samples or whether the same sample was measured repeatedly
☐	☒	The statistical test(s) used AND whether they are one- or two-sided<br>*Only common tests should be described solely by name; describe more complex techniques in the Methods section.*
☐	☒	A description of all covariates tested
☐	☒	A description of any assumptions or corrections, such as tests of normality and adjustment for multiple comparisons
☐	☒	A full description of the statistical parameters including central tendency (e.g. means) or other basic estimates (e.g. regression coefficient) AND variation (e.g. standard deviation) or associated estimates of uncertainty (e.g. confidence intervals)
☐	☒	For null hypothesis testing, the test statistic (e.g. *F*, *t*, *r*) with confidence intervals, effect sizes, degrees of freedom and *P* value noted<br>*Give P values as exact values whenever suitable.*
☒	☐	For Bayesian analysis, information on the choice of priors and Markov chain Monte Carlo settings
☒	☐	For hierarchical and complex designs, identification of the appropriate level for tests and full reporting of outcomes
☒	☐	Estimates of effect sizes (e.g. Cohen's *d*, Pearson's *r*), indicating how they were calculated

Our web collection on statistics for biologists contains articles on many of the points above.

Software and code

Policy information about availability of computer code

Data collection	Patient clinical and demographic data was collected using Progeny (v10.4.0.4) and Microsoft Excel (v16). Raw exome sequencing data (FASTQ format) was processed through a bioinformatics pipeline constructed using Seqliner (v0.7), in which reads were quality checked using FastQC (v0.11.2), trimmed using cutadapt (v1.5), aligned to the reference genome using BWA-MEM (v0.7.10), duplicates removed using Picard MarkDuplicates(v1.119), base quality scores recalibrated and indels realigned using GATK (v3.8) and variants called by GATK HaplotypeCaller and Platypus (v0.8.1).
Data analysis	Principal component analysis was performed in PLINK (v1.90). Variant (vcf format) filtering was performed in R (v3.5.2) with the tidyverse package (v1.2.1) installed, and the ranked output viewed in Microsoft Excel (v16). Graphs were plotted using GraphPad Prism (v8.1.1), and all statistical tests were performed in R or Prism. Integrative Genomics Viewer (v2.3.55) was used for manually reviewing variants in the pipeline-processed sequencing reads (bam format).

For manuscripts utilizing custom algorithms or software that are central to the research but not yet described in published literature, software must be made available to editors/reviewers. We strongly encourage code deposition in a community repository (e.g. GitHub). See the Nature Research guidelines for submitting code & software for further information.

Data

Policy information about availability of data

All manuscripts must include a data availability statement. This statement should provide the following information, where applicable:

- Accession codes, unique identifiers, or web links for publicly available datasets
- A list of figures that have associated raw data
- A description of any restrictions on data availability

The exome sequencing data have been deposited in the European Genome-Phenome Archive under the Study ID EGADxxxxxxxxxxx (https://ega-archive.org). Other datasets referenced during the study are available from the GnomAD (http://gnomad.broadinstitute.org) and COSMIC (https://cancer.sanger.ac.uk/cosmic) websites. All the other data supporting the findings of this study are available within the article and its supplementary information files and from the corresponding

author upon reasonable request. A reporting summary for this article is available as a supplementary information file.

Field-specific reporting

Please select the one below that is the best fit for your research. If you are not sure, read the appropriate sections before making your selection.

☒ Life sciences ☐ Behavioural & social sciences ☐ Ecological, evolutionary & environmental sciences

For a reference copy of the document with all sections, see nature.com/documents/nr-reporting-summary-flat.pdf

Life sciences study design

All studies must disclose on these points even when the disclosure is negative.

Sample size	Discovery sample size of 516 patients was the largest that could be recruited for exome sequencing who met the inclusion criteria (as detailed in the Methods), given the available funding and time constraints. Other gene discovery studies in this field (as referenced in the paper) have typically recruited much smaller samples, or have used large data sets (e.g. TCGA) that have been ascertained for other purposes.
Data exclusions	No data from the sample of 516 patients was excluded from analysis.
Replication	It was not practical or feasible to replicate the exome sequencing results given the resources available for this work, since this would necessitate recruitment and sequencing of another large sample of patients with similar characteristics to those described in the present study.
Randomization	Randomization was not relevant to or used in this study, since no intervention was applied and covariates could be controlled for through other means (i.e. case inclusion criteria and GnomAD control sub-population selection).
Blinding	Blinding was not relevant to or used in this study, since no intervention was applied and so unblinded analysis would not constitute a source of bias in this type of work.

Reporting for specific materials, systems and methods

We require information from authors about some types of materials, experimental systems and methods used in many studies. Here, indicate whether each material, system or method listed is relevant to your study. If you are not sure if a list item applies to your research, read the appropriate section before selecting a response.

Materials & experimental systems

n/a	Involved in the study	
☒	☐	Antibodies
☒	☐	Eukaryotic cell lines
☒	☐	Palaeontology
☒	☐	Animals and other organisms
☐	☒	Human research participants
☒	☐	Clinical data

Methods

n/a	Involved in the study	
☒	☐	ChIP-seq
☒	☐	Flow cytometry
☒	☐	MRI-based neuroimaging

Human research participants

Policy information about studies involving human research participants

Population characteristics	Women from Australia of predominantly Western European ethnicity (c.95%) with a confirmed or suspected prior diagnosis of high-grade serous (or similar) epithelial carcinoma arising in the ovary, fallopian tubes or peritoneum, aged between 24 and 88 at diagnosis, who have no germline pathogenic or likely pathogenic variants (as defined using the latest ACMG criteria) in the BRCA1 or BRCA2 genes following genetic testing.
Recruitment	All women were referred to and assessed at a specialist familial cancer clinic and tested for germline BRCA1 and BRCA2 genetic mutations (based on their personal and/or family history of cancer) in a diagnostic lab prior to recruitment. This may have resulted in an exclusion bias against women who might have been eligible for recruitment but were never referred to a familial cancer clinic for assessment, as well as against women who opted not to proceed with genetic testing. Western Europeans were over-represented in our cohort (c.95%), compared to their estimated prevalence in the Australian population (c. 85-90%), possibly reflecting a self-selection bias at point of testing and/or recruitment, and so it may be difficult to extrapolate our results to non-European ethnic groups.
Ethics oversight	This study was approved by the Peter MacCallum Cancer Centre Human Research Ethics Committee (approval nos. 11/50 and 09/29).

Note that full information on the approval of the study protocol must also be provided in the manuscript.
